# AuAg Nanoparticles Grafted on TiO_2_@N-Doped Porous Carbon: Improved Depletion of Ciprofloxacin under Visible Light through Plasmonic Photocatalysis

**DOI:** 10.3390/nano12152524

**Published:** 2022-07-22

**Authors:** Marta Jiménez-Salcedo, Miguel Monge, María Teresa Tena

**Affiliations:** 1 Department of Chemistry, University of La Rioja, C/Madre de Dios 53, E-26006 Logroño, Spain; marta.jimenez@aurea.unirioja.es; 2 Centro de Investigación en Síntesis Químicas (CISQ), University of La Rioja, C/Madre de Dios 53, E-26006 Logroño, Spain

**Keywords:** gold silver, titania, N-doped porous carbon, plasmonic, photocatalysis

## Abstract

TiO_2_ nanoparticles (NPs) were modified to obtain photocatalysts with different composition sophistication and displaying improved visible light activity. All of them were evaluated in the photodegradation of ciprofloxacin. The band gap of TiO_2_ NPs was successfully tailored by the formation of an N-doped porous carbon (NPC)-TiO_2_ nanohybrid through the pyrolysis of melamine at 600 °C, leading to a slight red-shift of the absorption band edge for nanohybrid NPC-TiO_2_ **1**. In addition, the in-situ formation and grafting of plasmonic AuAg NPs at the surface of NPC sheets and in close contact with TiO_2_ NPs leads to AuAg-NPC-TiO_2_ nanohybrids **2–3**. These nanohybrids showed superior photocatalytic performance for the degradation of ciprofloxacin under visible light irradiation, compared to pristine P25 TiO_2_ NPs or to AuAg-PVP-TiO_2_ nanohybrid **4** in which polyvinylpyrrolidone stabilized AuAg NPs were directly grafted to TiO_2_ NPs. The materials were characterized by transmission electron microscope (TEM), High Angle Annular Dark Field—Scanning Transmission Electron Microscopy—Energy Dispersive X-ray Spectroscopy HAADF-STEM-EDS, X-ray photoelectron spectroscopy and solid UV-vis spectroscopy. Moreover, the active species involved in the photodegradation of ciprofloxacin using AuAg-NCS-TiO_2_ nanohybrids were evaluated by trapping experiments to propose a mechanism for the degradation.

## 1. Introduction

Pharmaceuticals have been a milestone in medical care and eradication of diseases in recent decades but, at the same time, water quality has been worsened by continuous pharmaceuticals release into the environment. Consequently, pharmaceuticals have become emerging water pollutants. Among them, antibiotics stand out for their potential risk for many living beings to develop antibiotic resistance. Indeed, they pose a risk to the environment, ecology and the health of human beings [1], especially considering that wastewater treatment plants (WWTPs) were not a priori conceived to handle this kind of pollutants [2]. In this regard, ciprofloxacin (CIP), a widely used antibiotic, displays inert chemical bonds, which makes CIP very difficult to degrade by microorganisms and remains persistent in wastewater [3].

Advanced oxidation treatments have drawn great attention to tackle these issues. In particular, photocatalysis has gained increasing attention due to its non-toxicity, low-cost and non-polluting benefits to transform the bioactive molecule of pharmaceuticals into non-toxic by-products [4]. Semiconductor TiO_2_ NPs are one of the most widely used photocatalysts. However, its limited visible light activity, characteristic of large band gap semiconductors, and the high recombination rate of photoexcited electron-hole pairs are the main drawbacks that limit its use for practical applications. In this sense, semiconductor doping or heterojunction formation between semiconductors with different band gaps [5] constitute widely studied approaches that improve the photocatalytic performance of TiO_2_ NPs.

On the other hand, a very interesting class of emerging materials is nitrogen-doped porous carbon (NPC) materials [6]. These materials display interesting characteristics such as porous structures, nitrogen heteroatom active sites and the ability to act as catalyst or catalyst support in hydrogenation and oxidation reactions. In addition, the formation of N-doped graphitic conjugated π-structures provides improved photoinduced charge separation when these materials are combined with semiconductors [7]. NPCs can be synthesized, for instance, by the direct carbonization of nitrogen-rich precursors such as urea, melamine or aniline.

In addition, the use of plasmonic NPs displaying visible light harvesting properties is an interesting alternative to take advantage of the solar spectrum (ca. 43% visible light) and also prevent fast charge recombination when hybrid nanomaterials combining plasmonic nanoparticles and wide band gap semiconductors, such as TiO_2_, are designed. The visible light photoexcited electrons (hot electrons) can migrate from the metal to the conduction band (CB) of the semiconductor, which restrains the recombination of charges [8,9]. Localized surface plasmon resonance (SPR) of noble metal nanostructures could enhance solar light harvesting by additional mechanisms such as optical near-field enhancement and photothermal heating [8].

We wondered whether the combination of two approaches such as surface sensitization of TiO_2_ NPs in contact with NPC sheets and improved visible light absorption via plasmonic NPs would be an efficient strategy to overcome the lack of photocatalytic activity of TiO_2_ NPs in the visible range.

In this work, we propose a novel approach to tailor the visible light photocatalytic activity of a new type of hybrid nanostructures, by the purposeful combination of TiO_2_ semiconductor nanoparticles, nitrogen-doped porous carbon shells and plasmonic AuAg NPs. First, we have obtained an NPC-TiO_2_ nanohybrid, which, in a second step, has been decorated with in situ formed bimetallic AuAg plasmonic NPs. The bimetallic AuAg NPs were obtained by the mild decomposition of the organometallic complex [Au_2_Ag_2_(C_6_F_5_)_4_(OEt_2_)_2_]_n_, which has been previously used as a versatile precursor for the formation of plasmonic NPs, in the absence of stabilizing ligands or polymers [10,11,12,13,14]. The photocatalytic activity of these nanohybrids has been proved in the depletion of antibiotic ciprofloxacin. The photocatalytic activity of a similar system AuAg-PVP-TiO_2_ (**4**) (PVP = polyvinylpyrrolidone), in which the NPC component is not present, has been checked and compared with the ones bearing NPC sheets. In addition, we investigated the role of the active species involved in the photocatalytic mechanism through scavenging experiments. All the synthesized nanomaterials were characterized by transmission electron microscope (TEM), X-ray photoelectron spectroscopy (XPS) and solid UV-vis absorption spectroscopy.

## 2. Materials and Methods

### 2.1. Materials

Ciprofloxacin (CIP) (≥99%), titanium (IV) oxide (≥99.5%), polyvinylpyrrolidone (average mol wt 10,000) (PVP), tert-butanol, tetrahydrofuran (THF) and triethanolamine were obtained from Sigma-Aldrich (Schnelldorf, Germany). Formic acid (98%) for mass spectrometry was from Fluka Analytical. HPLC-grade acetonitrile was purchased from Scharlab (Barcelona, Spain). Ethyleneglycol and melamine (99%) were from Alfa Aesar (Kandel, Germany). Nylon syringe filters (13 mm, 0.22 µm) were from Scharlab (Barcelona, Spain).

The precursor [Au_2_Ag_2_(C_6_F_5_)_4_(OEt_2_)_2_]_n_ was prepared according to the literature [15].

### 2.2. Synthesis of Photocatalysts

NPC-TiO_2_ (**1**)

The NPC-TiO_2_ (**1**) nanohybrid was prepared by mixing a 70:30 weight ratio of P25 and melamine. Initially, 2 g of TiO_2_ (P25) and 0.86 g of melamine were suspended in 30 mL of distilled water at room temperature. The mixture was sonicated for 10 min, and it was evaporated to dryness under reduced pressure. The powder obtained was dried in an oven at 100 °C overnight. Secondly, thermal polycondensation and carbonization were carried out in alumina covered crucible to prevent sublimation at 600 °C for 3 h to obtain the nanohybrid. After that, the crucible was cooled at room temperature and the composite was crushed to obtain a homogeneous powder.

AuAg-NPC-TiO_2_ (**2**)

In total, 0.2116 g of **1** were sonicated for 30 min in 5 mL of ethylene glycol. Then, 1.5 mg (1.05 × 10^−3^ mmoL, 1% wt of metal content) of the bimetallic precursor [Au_2_Ag_2_(C_6_F_5_)_4_(OEt_2_)_2_]_n_ were added and the resulting mixture was also introduced in an ultrasound bath for 3 min. The suspension was stirred and heated under reflux at 130 °C, in darkness, for 15 min. After that, the obtained nanohybrid **2** was washed and centrifuged twice with distilled water, collected in ethanol and evaporated to dryness under reduced pressure.

AuAg-NPC-TiO_2_ (**3**)

Exactly 0.1047 g of **1** were sonicated for 30 min in 5 mL of ethylene glycol. Then, 1.5 mg (1.05 × 10^−3^ mmoL, 2% wt of metal content) of the bimetallic precursor [Au_2_Ag_2_(C_6_F_5_)_4_(OEt_2_)_2_]_n_ were added and the resulting mixture was also introduced in an ultrasound bath for 3 min. The suspension was stirred and heated under reflux at 130 °C, in darkness, for 15 min. After that, the obtained nanohybrid **3** was washed and centrifuged twice with distilled water, collected in ethanol, and evaporated to dryness under reduced pressure.

AuAg-PVP-TiO_2_ (**4**)

In order to prepare nanohybrid **4**, PVP stabilized AuAg were first synthesized. For this, 100 mg of [Au_2_Ag_2_(C_6_F_5_)_4_(OEt_2_)_2_]_n_ were dissolved in an excess of PVP (500 mg) with 80 mL of THF under argon atmosphere at 66 °C with reflux for 4 h, giving rise to a dark brown solid. The solvent was evaporated under vacuum and the isolated bimetallic nanoparticles were dissolved in 20 mL of distilled water and were placed in an ultrasound bath for 5 min. Finally, the compound was obtained after evaporation. The material was stored at 4 °C [16].

Then, 40 mg of the previously prepared AuAg-PVP-NPs were added to 10 mL of distilled water and the mixture was sonicated for 5 min. Then, 951.40 mg of TiO_2_ (Degussa P25) were added. The reaction mixture was stirred overnight at room temperature. The final product was washed and centrifuged three times with distilled water to remove the remaining PVP, collected in ethanol and evaporated to dryness under reduced pressure.

### 2.3. Characterization of Photocatalysts

Absorption UV−vis spectra of pressed powder samples diluted with silica were recorded on a Shimadzu (UV-3600 spectrophotometer with a Harrick Praying Mantis accessory, Kyoto, Japan). The absorption spectra were calculated from diffuse reflectance spectra and applying the Kubelka−Munk function. Transmission Electron Microscopy (TEM) samples were drop-casted from the ethanol dispersions (2–3 drops) to carbon-coated Cu grids. The corresponding TEM micrographs were acquired with a JEOL JEM 2100 microscope (Tokyo, Japan). In addition, High Angle Annular Dark Field—Scanning Transmission Electron Microscopy (HAADF-STEM) images were registered with a Tecnai F30 (ThermoFisher Scientific, Waltham, MA, USA) at a working voltage of 300 kV, coupled with a HAADF detector (Fischione, Export, PA, USA). In this operation mode, the intensity of the signal is proportional to the square of the atomic number (Z^2^), hence heavier elements such as gold or silver show a much brighter contrast than lighter elements, such as carbon or silicon. This is remarkably useful to localize metals in organic or light metal oxide matrixes. Furthermore, to analyze the chemical composition of the materials, X-ray Energy Dispersive Spectra (EDS) were registered in an EDAX detector or with an Ultim Max detector (Oxford, UK). XPS experiments were carried out with a Kratos AXIS Supra spectrometer (Manchester, UK), using a monochromatized Al Kα source (1486.6 eV) operating at 12 kV and 10 mA. Survey spectra were registered at analyzer pass energy of 160 eV, while narrow scans were acquired at constant pass energy of 20 eV and steps of 0.1 eV. The photoelectrons were detected at a take-off angle of F = 0° with respect to the surface normal. Basal pressure in the analysis chamber was less than 5 × 10^−9^ Torr. The spectra were obtained at room temperature. The binding energy (BE) scale was internally referenced to the C 1s peak (BE for C–C = 284.9 eV). The data treatment was carried out with the Casa XPS software using the specific relative sensitivity factor library that the software has for Kratos equipment. To calculate the atomic concentrations a Shirley-type background subtraction was used. The 4f and the 3d regions were used for Au and Ag, respectively. The RSF sensitivity factors used were Ti 2p = 2.001; C 1s = 0.278; O 1s = 0.780; N 1s = 0.477; Ag 3d = 5.987 and Au 4f = 6.25.

### 2.4. Photodegradation Procedure

In a typical procedure, 15.5 mg of the catalyst were suspended in 70 mL of a 4 µg mL^−1^ CIP aqueous solution in a 70-mL Schlenk glass reactor. Before the irradiation, the solution was treated in an ultrasound bath for 2 min approximately, and it was stirred for 60 min under dark conditions to reach adsorption/desorption equilibrium. Then, solutions were irradiated with visible LED light in a cooled lab-made setup. The assembly consists of four 10 W white light LED lamps (LED Engin, San Jose, CA, USA) placed equidistantly inside a cylindrical compartment. A constant temperature (25 °C) of the solution was maintained alongside the photodegradations thanks to a water recirculating coolant coil placed on the outside of the cylindrical compartment. During the degradation, the mixtures were stirred at 1200 rpm and 1.5 mL aliquots were collected at different times to monitor the reaction. All samples were filtered to remove the solid suspension of the catalyst and stored at 4 °C until analysis.

In order to evaluate active species responsible for the photodegradation, the CIP degradations were carried out under three different conditions: 10^−3^ M solution of tert-butanol and triethanolamine, to quench hydroxyl radicals (·OH) and photogenerated holes (h+), respectively and under N_2_ atmosphere to quench superoxide radicals (·O_2_^−^) [17,18,19,20]. All the experiments were carried out at in ultrapure water at natural pH.

### 2.5. Photodegradation Analysis

The concentration of ciprofloxacin during the photodegradations was monitored by a high-performance liquid chromatography system. The equipment was an Agilent modular 1100/1200 liquid chromatography system (Agilent Technologies, Santa Clara, CA, USA) equipped with a G1379A degasser, a G1311A HPLC quaternary pump, a G1329A autosampler and a G1315D diode array detector. A Phenomenex Luna^®^ LC C18 100 Å (5 µm particle size, 150 mm × 4.6 mm i.d.) column was used for the separation of compounds. The mobile phase was a 75:25 mixture of 0.1% formic acid in methanol and 0.1% formic acid aqueous solution. Injection volume was 20 µL, the flow rate was 1.0 mL min^−1^ and the separation was performed at room temperature. Detection wavelength was 280 nm.

## 3. Results and Discussion

### 3.1. Synthesis and Characterisation of Photocatalysts

**Scheme 1:**
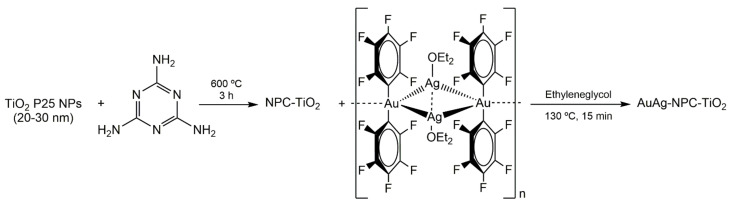
Synthesis of NPC-TiO_2_ nanohybrid **1** and AuAg-NPC-TiO_2_ nanohybrids **2–3** by reduction of complex [Au_2_Ag_2_(C_6_F_5_)4(OEt_2_)_2_]_n_ in ethylene glycol (EG) at 130 °C.

The first aim of this study was the design of a facile and straightforward approach for the preparation of AuAg-NPC-TiO_2_ nanohybrids (**2–3**) with enhanced photocatalytic properties. The synthesis was a two-step process (see Scheme 1).

First, nitrogen-doped porous carbon was formed in the presence of TiO_2_ NPs by the carbonization of melamine (initial 30:70 melamine:TiO_2_ weight ratio) at 600 °C, leading to nanohybrid NPC-TiO_2_ **1**. Then, the organometallic compound [Au_2_Ag_2_(C_6_F_5_)_4_(OEt_2_)_2_]_n_ (1% wt. of added Au-Ag content in the precursor (**2**) or 2% wt (**3**)) was decomposed under mild reducing conditions (130 °C, 15 min in ethylene glycol) on the surface of NPC-TiO_2_ nanohybrid.

**Figure 1:**
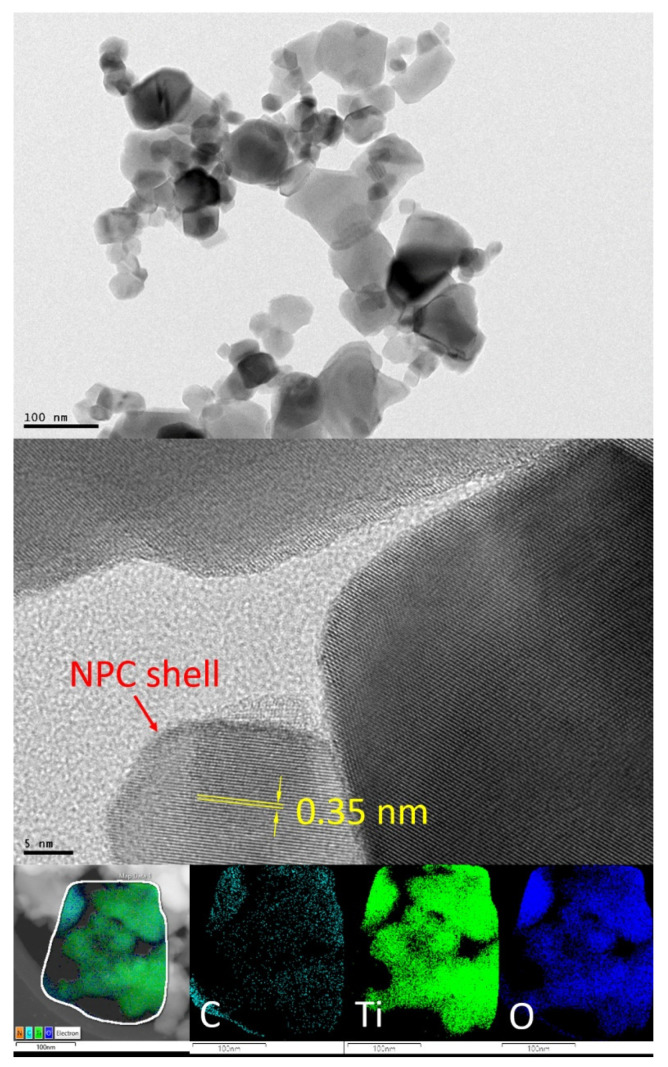
TEM image of NPC-TiO_2_ **1** (**up**); HRTEM image of NPC-TiO_2_ **1** (**middle**); HAADF-STEM-EDS image of NPC-TiO_2_ nanohybrid **1** (**bottom-left**). The images for the EDS elemental mapping for elements C, Ti and O: bottom-left image display all elements mapping at a time, while the rest of images display the individual elemental maps.

Figure 1 depicts the TEM, HRTEM and HAADF-STEM-EDS micrographs of a sample of NPC-TiO_2_ nanohybrid **1**, showing the presence of sheets of NPCs of ca. 100 nm size covering TiO_2_ NPs of ca. 15–25 nm size.

A closer inspection of the TiO_2_ NPs through HRTEM clearly shows the formation of an amorphous layer in the border of monocrystalline TiO_2_ NPs with 0.35 nm of interplanar spacing assigned to the (101) plane of anatase phase in P25 TiO_2_. The EDS analysis of a selected area depicted in Figure 1, shows the homogeneous distribution of C, Ti and O along the whole selected area, being more abundant in the presence of Ti and O. The presence of N is not detected through EDS analysis, which points to the presence of very small amounts of this element in the nanohybrids, if present. This EDS analysis confirms the presence of both NPC and TiO_2_ nanomaterials at the same position, keeping a main composition of TiO_2_, in agreement with the lower added weight amount of melamine.

The decomposition of [Au_2_Ag_2_(C_6_F_5_)_4_(OEt_2_)_2_]_n_ over nanohybrid **1** leads to the formation of AuAg-NPC-TiO_2_ nanohybrids (**2–3**). This synthetic approach allows the direct stabilization of small-size Au-Ag NPs stabilized by the NPC surfaces, probably by the interaction with the N-donor groups of this material, as previously reported for NPC-Pd NPs [21], avoiding the use of additional polymers or ligands as stabilizing agents for the plasmonic NPs (see Scheme 1).

**Figure 2:**
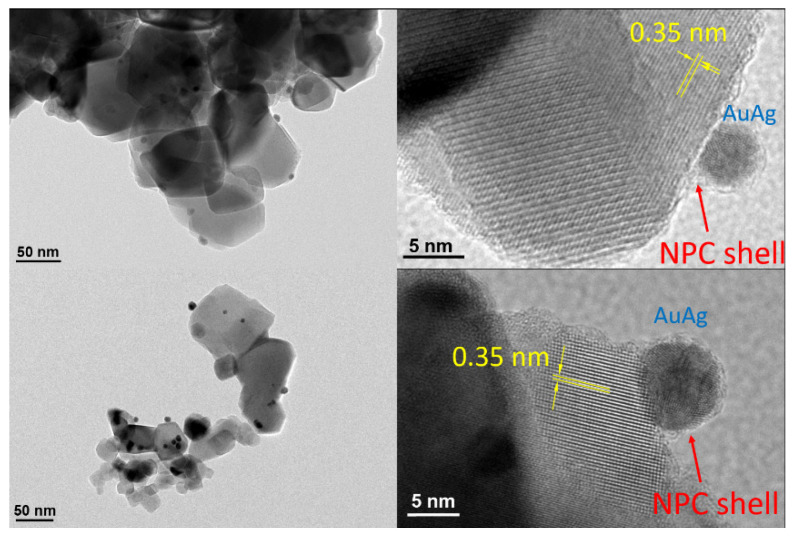
TEM and HRTEM images of Au-Ag-NCS-TiO_2_ nanohybrids **2** (**up**) and **3** (**bottom**).

Indeed, the direct reduction of the Au(I)-Ag(I) organometallic precursor in the presence of TiO_2_ NPs without the concurrence of additional stabilizing polymers or ligands leads to the formation of bulk metals. Figure 2 displays the TEM and HRTEM analysis of samples of nanohybrids **2** and **3**. The TEM images show the presence of small-sized spherical NPs of 5.3 ± 1.2 nm (**2**) or 9.5 ± 1.9 nm (**3**) (see size histograms and TEM images for nanohybrid AuAg-PVP-TiO_2_ **4** in Supplementary Materials, Figures S1 and S2, respectively), The HRTEM images display polycrystalline Au-Ag nanoparticles for **2** and **3**. TiO_2_ NPs display constant d = 0.35 nm values in agreement with the growth along the (101) facet and NPC appears as nanosheets and shells surrounding the bimetallic NPs. In many cases, the AuAg NPs appear on the surface of TiO_2_ NPs.

**Figure 3:**
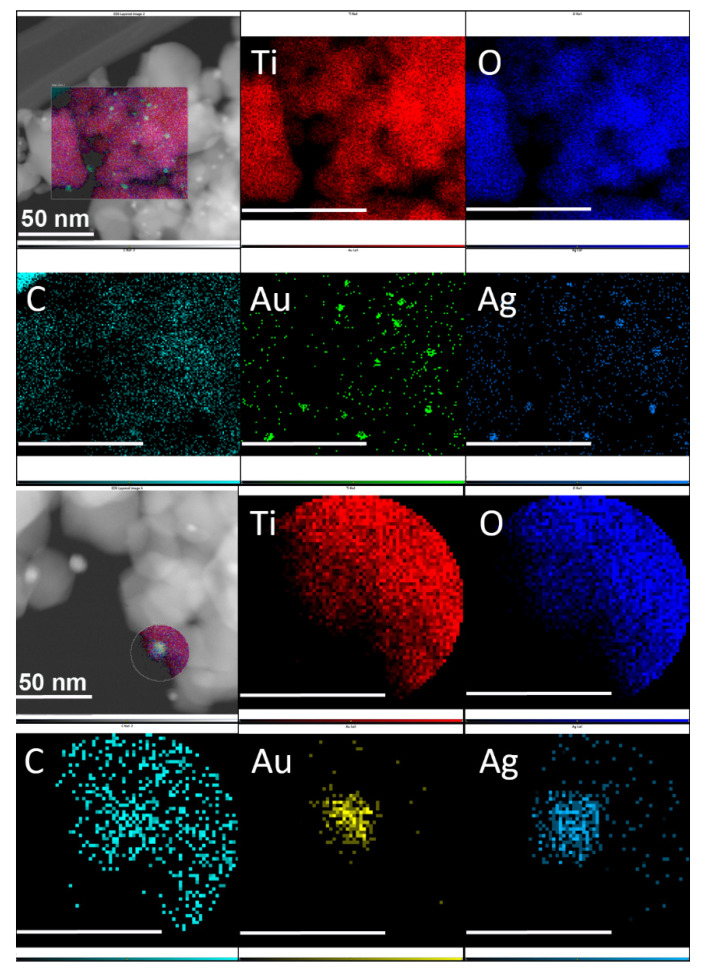
Lower-magnification (**up**) and detailed (**bottom**) HAADF-STEM images of AuAg-NPC-TiO_2_ nanohybrid **2**. EDS elemental mapping images for elements Ti, O, C, Au and Ag: larger images display all elements mapping together, while the rest of the images display the individual Ti, O, C, Au and Ag elemental maps, respectively (bar corresponds to 50 nm for the lower magnification images and 25 nm for the detailed images).

The HAADF-STEM-EDS study of Au-Ag-NPC-TiO_2_ nanohybrid **2** was performed to confirm the pursued composition and distribution of each component of the nanohybrid. The larger images in Figure 3 depict HAADF-STEM images with low (upper image) or high (bottom image) magnification of **2**. These images show small NPs with high Z-contrast and TiO_2_ NPs covered with NPC nanosheets (see Supplementary Materials). The EDS analysis of selected regions displays a main homogeneous composition of Ti and O, together with a lesser contribution from C, but also extended in the same region. A minor composition of Au and Ag is located at similar places, corresponding with the presence of AuAg alloyed NPs. This is particularly detailed in the high-magnification EDS images in Figure 3, where a bimetallic AuAg NP appears at the edge of a region with NPC-TiO_2_ composition.

**Figure 4:**
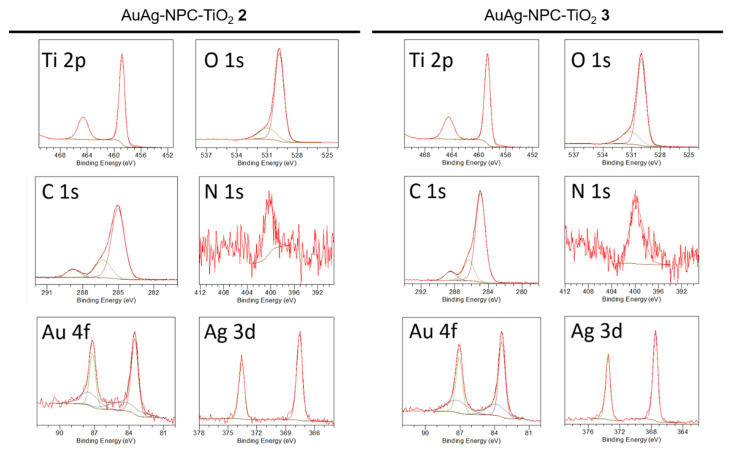
Narrow XPS spectra for Ti 2p, O 1s, C 1s, N 1s, Au 4f and Ag 3d for Au-Ag-NPC-TiO_2_ **2** and **3**.

The elemental composition and chemical states on the surface of nanohybrids **1–3** were studied through XPS. Figure 4 shows the Ti 2p, O 1s, C 1s, N 1s, Au 4f and Ag 3d narrow spectra for **2** and **3**. The corresponding survey spectra for **1**-**3** and narrow XPS spectra for **1** are included in the Supplementary Materials (see Figures S3 and S4 and Tables S1 and S2).

Intense peaks corresponding to Ti and O are observed in the survey XPS spectrum of the nanohybrids **1–3**. Smaller peaks are assigned to C, and in the case of f **2** and **3**, Au and Ag peaks can also be found, whereas an almost negligible peak corresponding to N is detected for all nanohybrids. The % atomic composition (narrow spectra) of Ti is 22.78 (**1**), 21.98 (**2**) and 21.52% (**3**), whereas for O is 55.95 (**1**), 56.50 (**2**) and 56.77% (**3**), being these elements the most abundant in the samples, decreasing as the metal content increases (see below). The C composition is 21.11 (**1**), 20.98 (**2**) and 20.84% (**3**) and the one found for N is only 0.16 (**1**), 0.32 (**2**) and 0.47% (**3**), confirming the formation of nitrogen-doped carbon material from melamine, due to the high-temperature carbonization conditions that lead to the nitrogen burnt off from the nanohybrid surface. This result is similar to other previously reported when N-rich molecules such as melamine and TiO_2_ are pyrolyzed together [7,22]. Minor atomic compositions of Ag (0.13 (**2**) and 0.22% (**3**)) and Au (0.09 (**2**) and 0.17% (**3**)) are also found, which corresponds to % weight compositions of 0.62 (**2**), 1.05 (**3**) and 0.79 (**2**), 1.49% (**3**), respectively, slightly higher than the 1% wt of added metal in the organometallic precursor. This is probably due to the loss of some NPC-TiO_2_ **1** nanoparticles in the preparation process of **2** and **3** nanohybrids.

The narrow XPS spectrum for the Ti 2p region for **2** and **3** displays a doublet at 458.8 and 464.6 (**2**) or 458.7 and 464.5 eV (**3**) with a separation of 5.7 eV that is assigned to the presence of TiO_2_ in the sample [23]. Ti-N and Ti-C bonds are discarded, ruling out the N or C doping in the TiO_2_ crystal lattice. The presence of TiO_2_ is further confirmed by the narrow spectrum found for O 1s for **2** and **3** with binding energies of 529.8 and 530.9 (**2**) or 530.0 and 531.1 eV (**3**), which are assigned to oxygen anions in the TiO_2_ lattice (Ti–O) and to surface OH groups, respectively.

The C 1s region for **2** and **3** is fitted into three main peaks at 285.0, 286.3 and 288.8 (**2**) or 285.0, 286.3 and 288.5 eV (**3**). The lowest binding energy peak at 285.0 eV corresponds to single C-C/C-H binding; since the presence of N in the sample is almost negligible, the deconvoluted peaks at 286.3 and 288.5 eV could be assigned to C-O and C=O bonds, respectively [24]. These peaks would confirm the interaction between NPC and TiO_2_ and rule out the TiO_2_ doping. The N 1s peak is very weak and appears at 400.4 (**2**) or 399,4 eV (**3**), corresponding to tertiary N-(C)3 binding, being absent the peak at ca. 398 eV corresponding to N=C-N bonding in triazine groups of g-C_3_N_4_, in agreement with a high degree of carbonization of melamine and N-doping.

The narrow XPS spectrum for the Au 4f region for **2** and **3** is depicted in Figure 4. The experimental signals are fitted to two spin-orbit doublets of different intensities, but equally separated in energy (ca. 3.7 eV). The most intense doublet at 83.5 eV and 87.2 eV (**2**) or 83.4 and 87.1 eV (**3**) is attributed to metallic gold. The presence of Au(I) surface atoms can be assigned to a weaker intensity doublet at 84.4 and 87.4 (**2**) or 83.8 and 87.2 eV (**3**) can be attributed to the presence of Au(I) and Au(III) oxidation states. The positive oxidation state for Au atoms is usually found when sub-10 nm size particles are grafted onto C-based surfaces such as g-C_3_N_4_ or graphene [25].

The narrow XPS spectrum for the Ag 3d region for **2** and **3** shown in Figure 4 displays two signals that are fitted to one spin-orbit doublet with a separation in the energy of ca. 6.0 eV, characteristic of the silver 3d region at 367.6 and 373.6 (**2**) or 367.5 and 373.5 eV (**3**). Considering the energy maxima of the doublet these signals are attributed to metallic silver [26].

The low binding energy values for Au 4d and Ag 3d, usually between 84.3-84.5 eV and 368.1-368.3 eV, respectively, could be ascribed both to AuAg alloy formation and to AuAg NPs-TiO_2_ substrate interactions, as previously reported [27,28].

**Figure 5:**
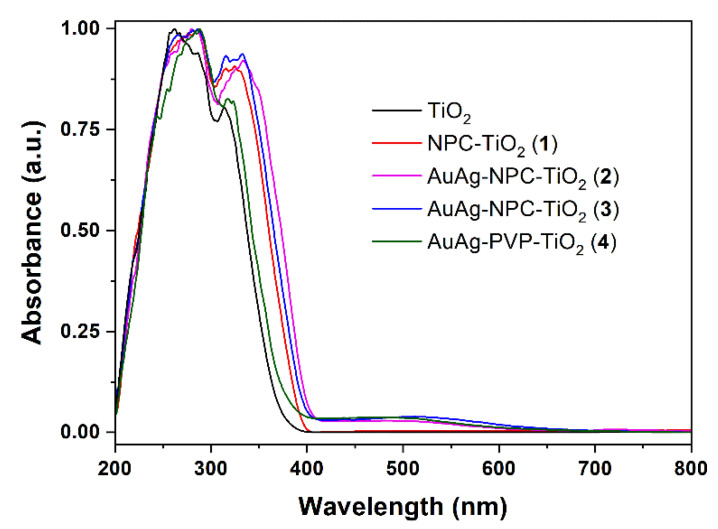
Solid UV-Vis spectra for pristine TiO_2_ and nanohybrids **1–4**.

Figure 5 shows the solid UV-vis absorption spectra of the samples. Comparing TiO_2_ and AuAg-PVP-TiO_2_ (**4**) spectra, it is observed that the addition of PVP-stabilized bimetallic nanoparticles slightly red-shifts the absorption band edge of TiO_2_ and a plasmonic absorption band in the visible region at ca. 495 nm appears. The formation of the NPC layers on TiO_2_ NPs in nanohybrid **1** produces an important red-shift of the absorption band edge of TiO_2_. Additionally, when nanohybrid **1** is grafted with plasmonic AuAg nanoparticles, an even more pronounced band edge red-shift is produced together with the appearance of a plasmonic absorption at 497 nm (**2**) and 512 nm (**3**), in the 400–650 nm range.

The corresponding Tauc plots (Figure S5 in Supplementary Materials) display the values of the TiO_2_ band-gap in the nanohybrids. Further, the metal-semiconductor heterojunction produces a slight decrease in the TiO_2_ band gap from 3.10 in TiO_2_ NPs to 2.99 eV in TiO_2_-PVP-AuAg (**4**). The inclusion of NPC layers on TiO_2_ NPs also produces a band-gap decrease to 2.97 eV, whereas the inclusion of AuAg NPs in nanohybrid **1** leads to even narrower band-gaps of 2.92 (**2**) and 2.88 eV (**3**), respectively. These results confirm that the presence of both the NPC layers and the plasmonic AuAg NPs favors the band-gap reduction of TiO_2_. In addition, the plasmonic absorption produced by the presence of tiny amounts of AuAg NPs increases the visible light harvesting ability of these systems and the boosting of the LSPR effects.

### 3.2. Photocatalytic Activity

**Figure 6:**
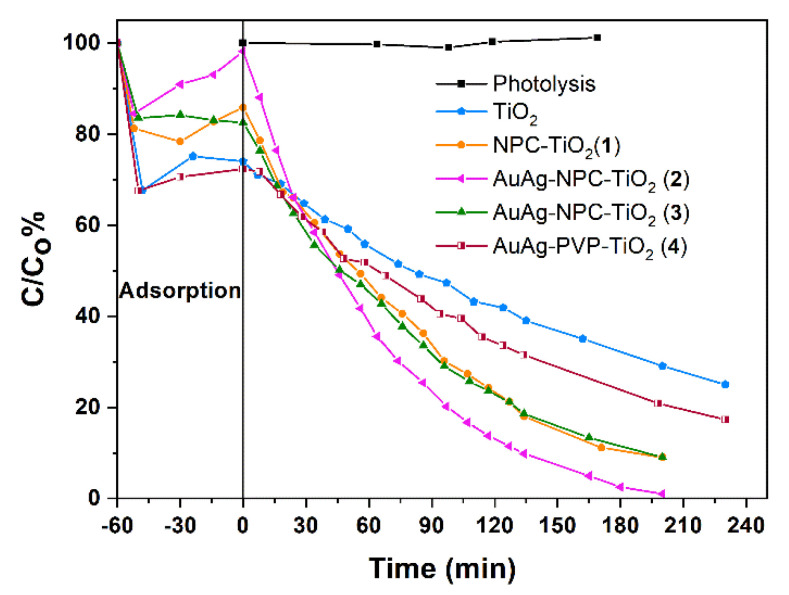
Evolution of ciprofloxacin concentration during its degradation with TiO_2_ and nanohybrids **1–4**, under visible LED light.

Figure 6 shows the evolution of the concentration of ciprofloxacin (CIP) under visible light in the presence of the previously described photocatalysts. The adsorption of CIP decreases in nanomaterials that contain NPC shells, whereas the adsorption with TiO_2_ and nanohybrid (**4**) is around 30%.

CIP degradation in the absence of photocatalyst (photolysis) was negligible. About 70% of CIP was removed within 230 min of irradiation after the addition of TiO_2_ (P25). A partial absorption in the visible light region for TiO_2_ was also observed by other authors [5]. In addition, the white LED light used displays a relative spectral power in the 400–800 nm range (maxima at ca. 460 and 550 nm), being the higher energy limit very close to the TiO_2_ band-gap energy. The photodegradation of CIP is enhanced when plasmonic nanoparticles are grafted on TiO_2_ which corresponds to nanohybrid **4**.

As can be seen in Figure 6, the depletion rates achieved with nanohybrids NPC-TiO_2_ (**1**), AuAg-NPC-TiO_2_ (**2**) and AuAg-NPC-TiO_2_ (**3**) were higher than the ones obtained for pristine TiO_2_ and AuAg-PVP-TiO_2_ (**4**). Total CIP degradation was achieved with catalyst **2** within 200 min of irradiation. Moreover, the degradation rates observed with nanohybrids **1** and **3** were similar to each other and were lower than the one obtained with **2**, pointing out that a higher percentage of metal did not enhance the photodegradation rate. Furthermore, the size of the NPs might play a role in the degradation effectiveness, since the mean size of the NPs in the nanohybrid **2** was smaller than the one for **3**, displaying in the former a higher surface/volume ratio.

All the degradations followed pseudo-first kinetic orders (see Figure S6 in Supplementary Materials) with constants values of: 0.0046, 0.0064, 0.012, 0.011, 0.018 min^−1^ for TiO_2_, Au-Ag-PVP-TiO_2_ (**4**), NPC-TiO_2_ (**1**), Au-Ag-NPC-TiO_2_ (**3**) and Au-Ag-NPC-TiO_2_ (**2**), respectively.

### 3.3. Mechanism of Photocatalytic Activity

**Figure 7:**
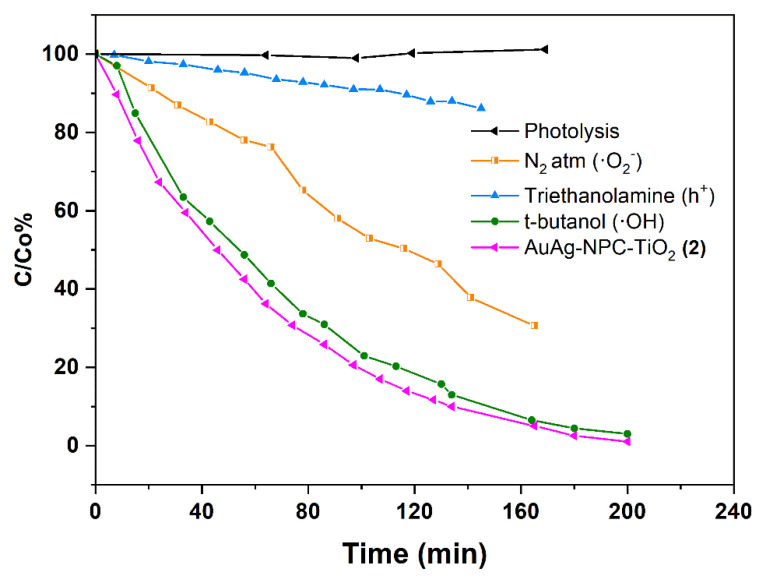
Evolution of CIP concentration during photocatalytic degradation with **2** under visible light irradiation and effect of scavengers.

During CIP degradation experiments, reactive oxygen species are generated upon visible light irradiation, and they are responsible for the CIP depletion. In order to study the photocatalytic mechanism with the catalyst AuAg-NPC-TiO_2_ (**2**) and identify which reactive species play a significant role in the photodegradation of CIP, three trapping experiments were performed. Figure 7 shows how CIP depletion rates vary when the degradations are carried out in the presence of tert-butanol or triethanolamine and under an N_2_ atmosphere. The addition of triethanolamine greatly decreased, by around 90%, the degradation efficiency of CIP, suggesting that the holes (h^+^) are the main reactive species in the photodegradation process. Moreover, the degradation was partially inhibited under the N_2_ atmosphere and, consequently, superoxide radicals (·O_2_^−^) also play an important role. However, slight suppression in the degradation of CIP is observed when tert-butanol is added, which means that the hydroxyl radicals (·OH) had little contribution to the CIP photodegradation process with the photocatalysts **2**. These results agree with a recent report on the photocatalytic degradation of Rhodamine B using Au/TiO_2_ network-like nanofibers as photocatalysts [29].

**Figure 8:**
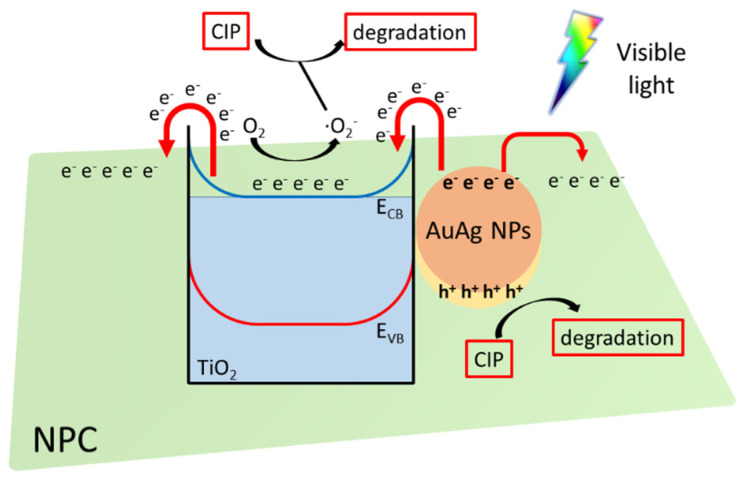
Schematic representation of the mechanism of degradation of CIP through the plasmonic hot-electron injection from AuAg NPs to the CB of TiO_2_ and further charge-carrier separation in NPC shells.

Following the observations of the scavenger effects, the improved photocatalytic activity of nanohybrid **2** can be related to the visible light LSPR absorption of the bimetallic AuAg NPs grafted at the surface of TiO_2_ NPs and stabilized with NPC shells. The plasmonic absorption enables the hot-electron injection from the alloyed NPs to the conduction band of the TiO_2_ semiconductor. This hot-electron injection produces an electron-hole pair formation and further charge carrier separation [7]. In addition, the presence of N-doped porous carbon (NPC) layers strongly contributes to the charge carrier separation. The photogenerated electrons are able to react with absorbed O_2_, leading to superoxide radicals (·O_2_^−^) through a reduction process. A schematic representation of the mechanism of photocatalysis is depicted in Figure 8.

The almost negligible role played by hydroxyl radicals agrees with the unfavorable formation of photogenerated electrons upon TiO_2_ excitation. Indeed, the semiconductor band-gap is slightly larger than the higher energy component of visible light, ruling out the formation of holes (h^+^) at the valence band of TiO_2_ (1.65 eV vs. NHE), which would display a favorable potential for the formation of the ·OH radicals from OH^−^ groups (·OH/OH^−^ potential is 2.38 eV, vs. NHE) or from H_2_O (·OH/H_2_O potential is 2.72 eV, vs. NHE).

## 4. Conclusions

We have developed a straightforward and efficient approach for the synthesis of N-doped porous carbon-titania nanohybrids displaying enhanced photocatalytic activity towards the degradation of CIP under visible light when this material is grafted with plasmonic AuAg alloyed NPs. The presence of AuAg nanoparticles provides the possibility of hot-electron injection to the CB of TiO_2_ upon visible light plasmonic absorption thanks to an effective metal-semiconductor interface formation. The charge-carrier separation is clearly enhanced by the presence of NPC layers and improves the photocatalytic efficiency of the nanohybrid photocatalysts towards the degradation of CIP, which is a persistent antibiotic in water.

## Data Availability

The data presented in this study are available on request from the corresponding authors.

## Supplementary Materials

The following are available online at https://www.mdpi.com/article/10.3390/nano12152524/s1, Figure S1: Size histograms of nanohybrids **2**–**4**. Figure S2: TEM images of nanohybrid AuAg-PVP-TiO_2_ **4**. Figure S3: Survey XPS spectrum for nanohybrids **1**–**3**. Figure S4: Narrow XPS spectrum for Ti 2p, O 1s, C 1s and N 1s in nanohybrid **1**. Figure S5: Tauc plots for **1** (up-left), **2** (up-right), **3** (bottom-left) and **4** (bottom-right). Figure S6: Kinetics of the photocatalysts **1**–**4** under visible light. The fitting results are represented assuming a pseudo-first order reaction. Table S1: Deconvolution of the XPS high-resolution peaks (binding energy in eV) for nanohybrids **1**–**3**. Table S2: At% and wt% data for nanohybrids **1**–**3**.
