# Supplementary material for: AuAg Nanoparticles Grafted on TiO2@N-Doped Porous Carbon: Improved Depletion of Ciprofloxacin under Visible Light through Plasmonic Photocatalysis
Source: Nanomaterials (Basel). 2022 Jul 22;12(15):2524. doi: 10.3390/nano12152524 (PMC9329855; doi:10.3390/nano12152524)
Supplement: Supplementary file 1 [file nanomaterials-12-02524-s001.zip › nanomaterials-1806102-supplementary.pdf]

nanomaterials

MDPI

Supplementary Materials

AuAg Nanoparticles Grafted on TiO_2@N-Doped Porous Carbon: Improved Depletion of Ciprofloxacin under Visible Light through Plasmonic Photocatalysis

Marta Jiménez-Salcedo [1], Miguel Monge [1,2,*] and María Teresa Tena [1,*]

[1] Department of Chemistry, University of La Rioja, C/Madre de Dios 53, E-26006 Logroño, Spain; marta.jimenez@aurea.unirioja.es

[2] Centro de Investigación en Síntesis Químicas (CISQ), University of La Rioja, C/Madre de Dios 53, E-26006 Logroño, Spain

* Correspondence: miguel.monge@unirioja.es (M.M.); maria-teresa.tena@unirioja.es (M.T.T.)

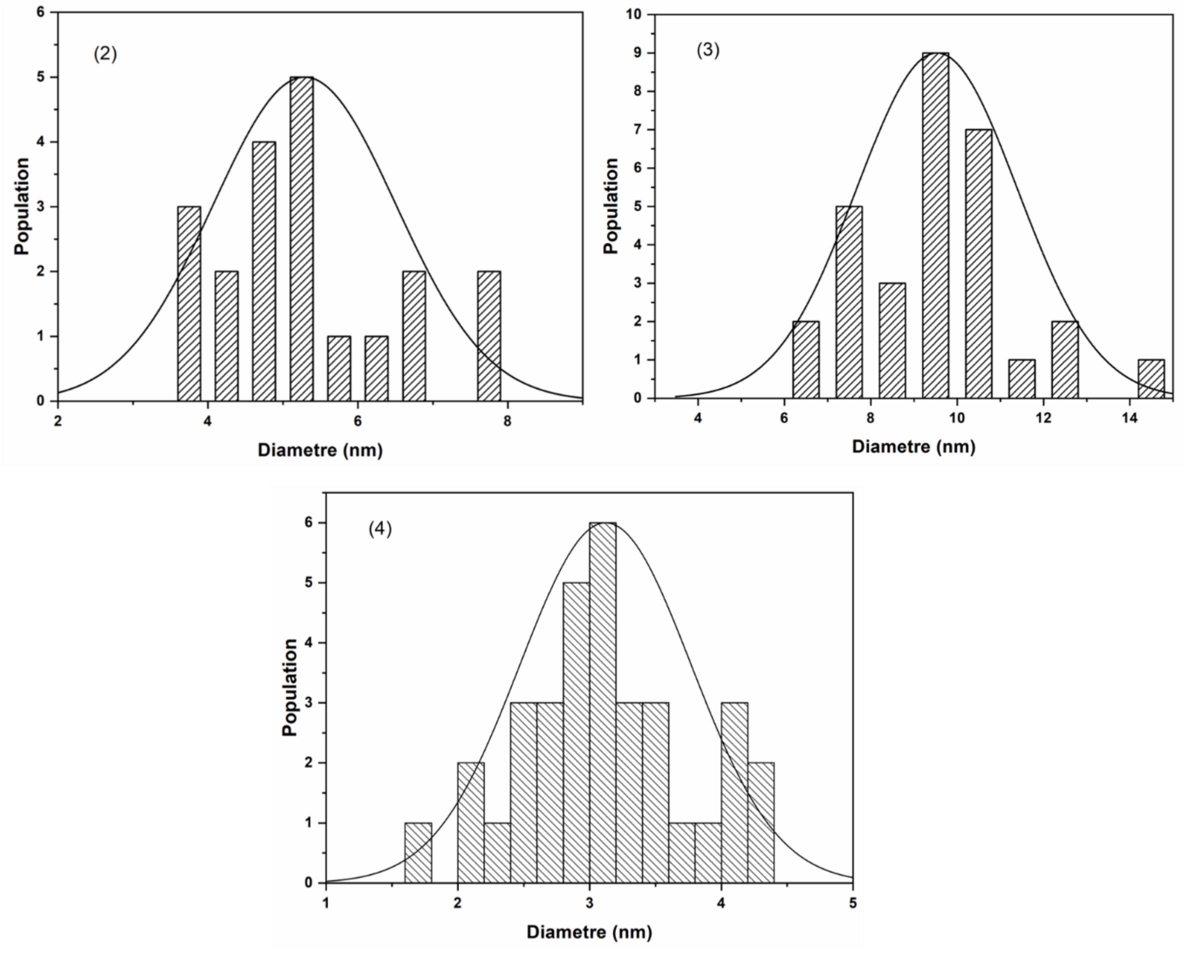

Figure S1. Size histograms of nanohybrids **2–4.**

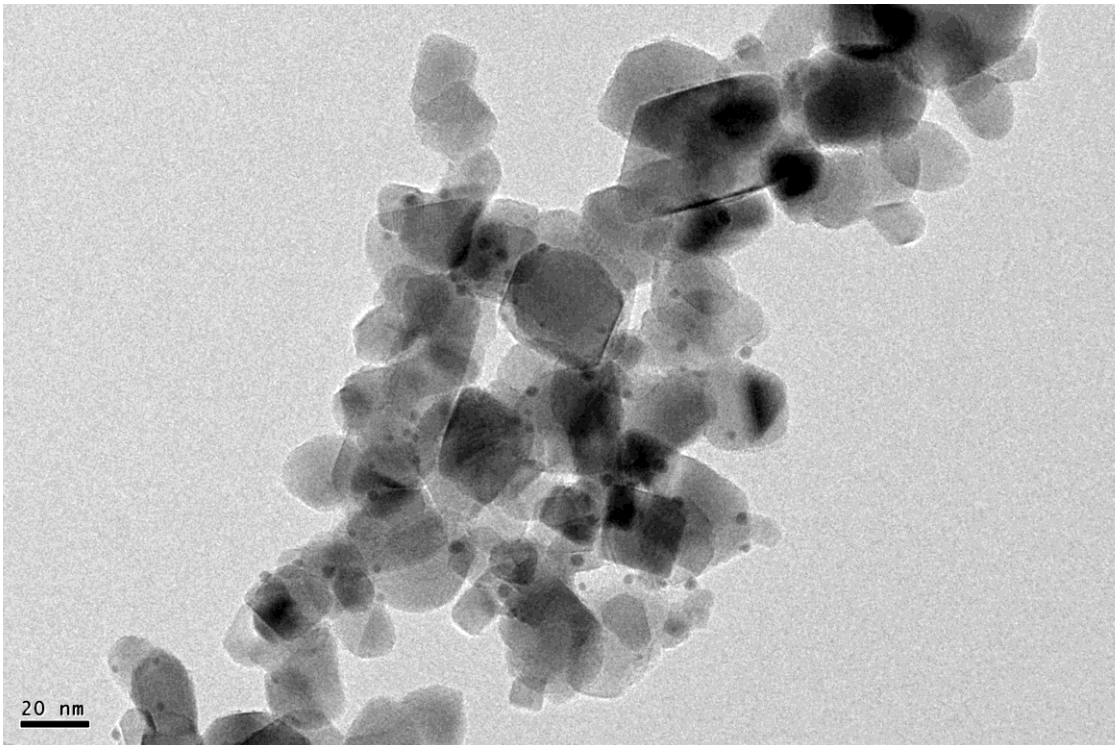

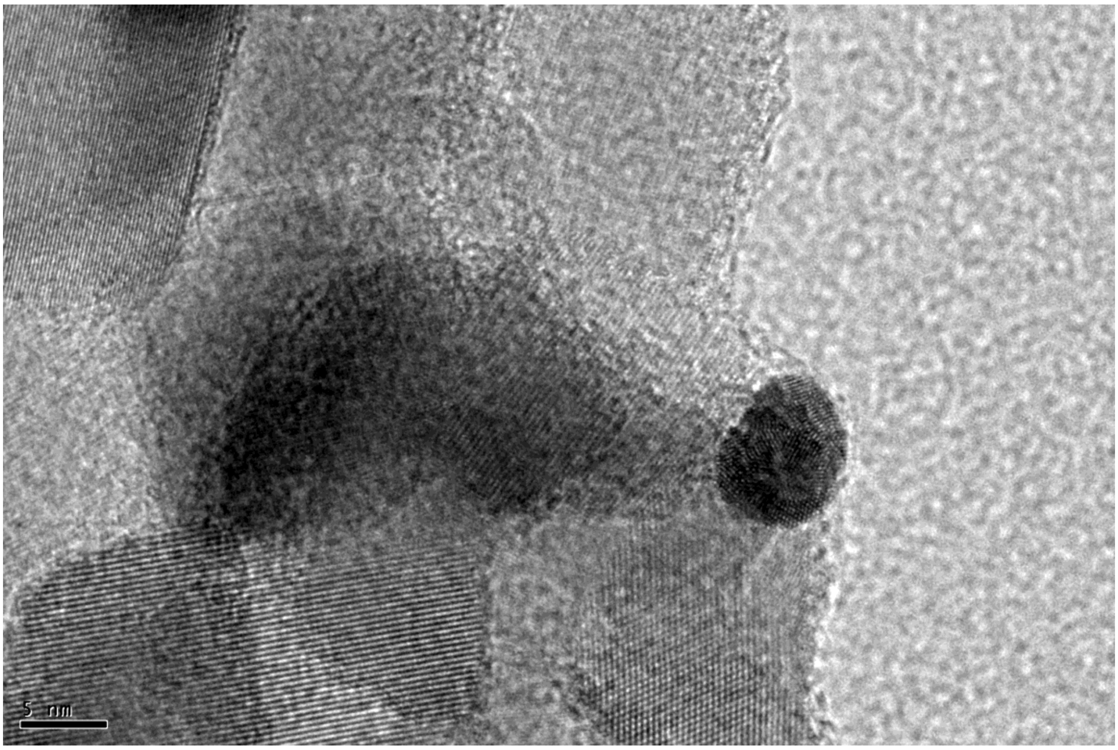

Figure S2. TEM images of nanohybrid AuAg-PVP-TiO_2 **4**.

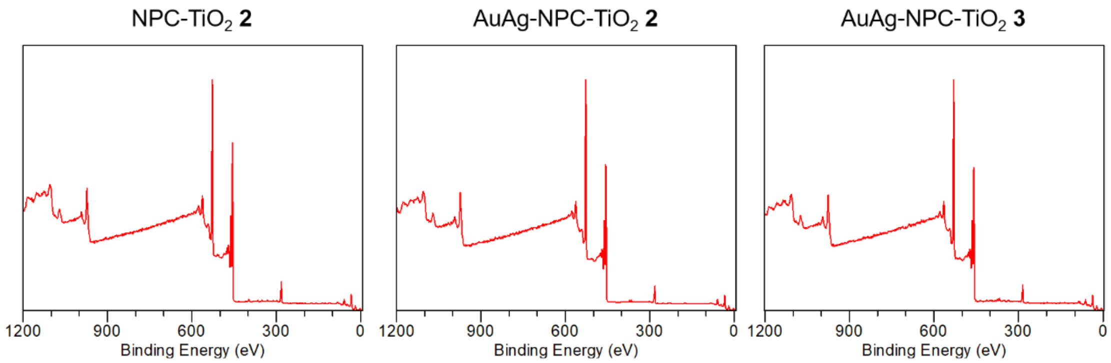

Figure S3. Survey XPS spectrum for nanohybrids **1–3**.

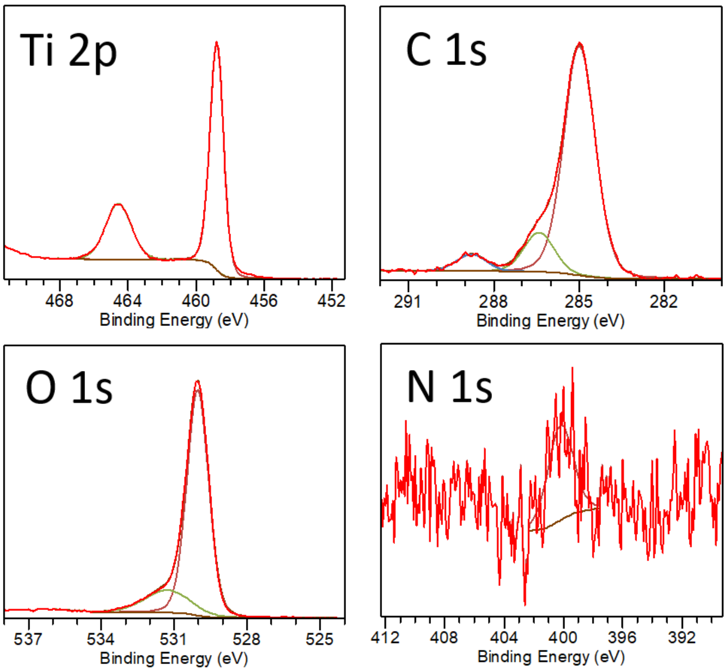

Figure S4. Narrow XPS spectrum for Ti 2p, O 1s, C 1s and N 1s in nanohybrid **1**.

Table S1. Deconvolution of the XPS high-resolution peaks (binding energy in eV) for nanohybrids **1–3.**

	**C 1s**	**N 1s**	**Ti 2p**	**O 1s**	**Au 4f**	**Ag 3d**
**1**	285.0, 286.4, 288.8	400.2	458.8, 464.6	530.0, 531.3	-	-
**2**	285.0, 286.3, 288.8	400.4	458.8, 464.6	529.8, 530.9	83.5, 84.4, 87.2, 87.4	367.6, 373.6
**3**	285.0, 286.3, 288.5	399.4	458.7, 464.6	530.0, 531.3	83.4, 83.8, 87.1, 87.2	367.5, 373.5

Table S2. At% and wt% data for nanohybrids **1–3.**

	**C 1s**	**N 1s**	**Ti 2p**	**O 1s**	**Au 4f**	**Ag 3d**
**1**						
**at.%**	21.11	0.16	22.78	55.95	-	-
**wt.%**	11.31	0.10	48.66	39.93	-	-
**2**						
**at.%**	20.98	0.32	21.98	56.50	0.09	0.13
**wt.%**	11.23	0.20	46.89	40.27	0.79	0.62
**3**						
**at.%**	20.84	0.47	21.52	56.77	0.17	0.22
**wt.%**	11.11	0.29	45.74	40.31	1.49	1.05

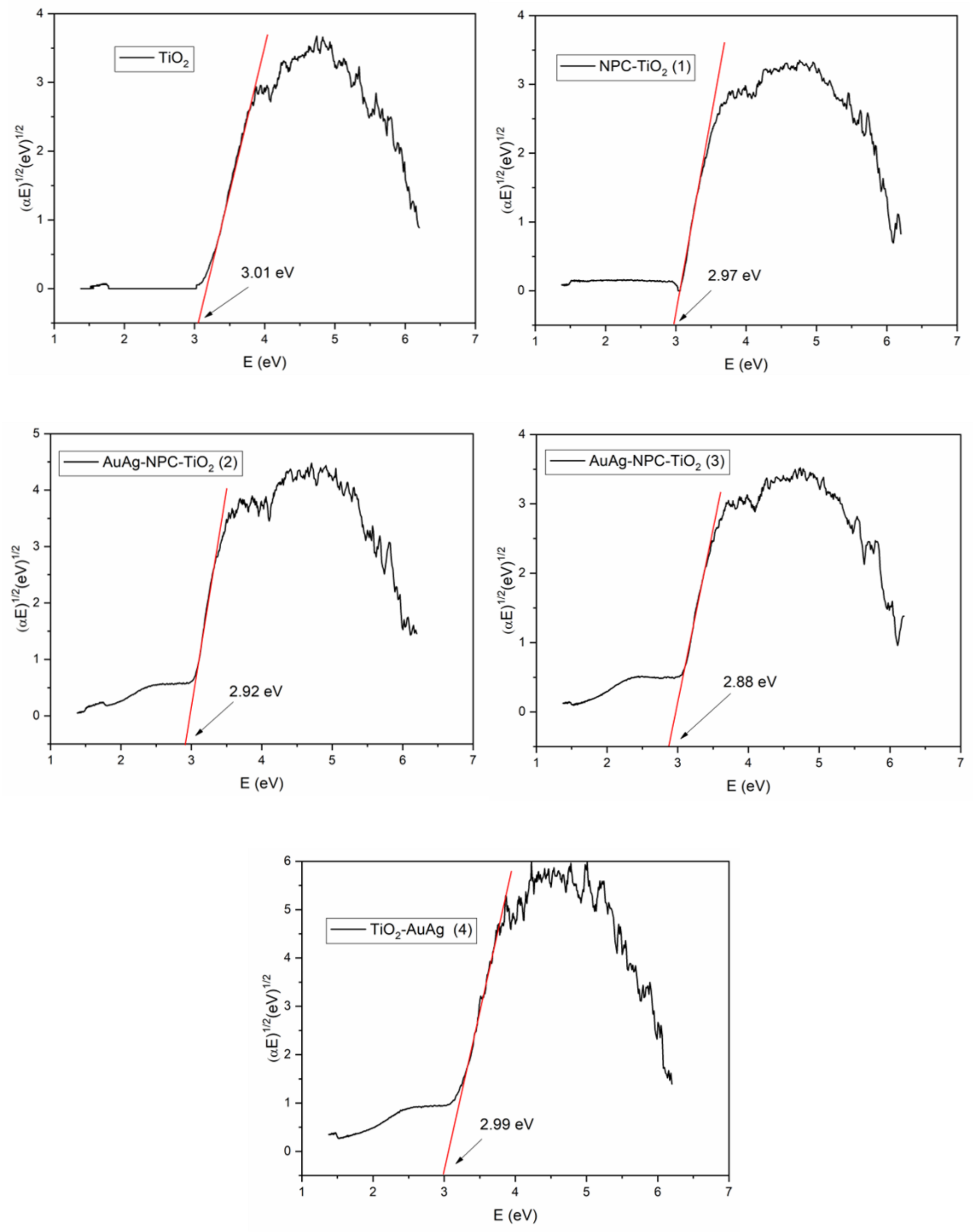

Figure S5. Tauc plots for 1 (up-left), 2 (up-right), 3 (bottom-left) and 4 (bottom-right).

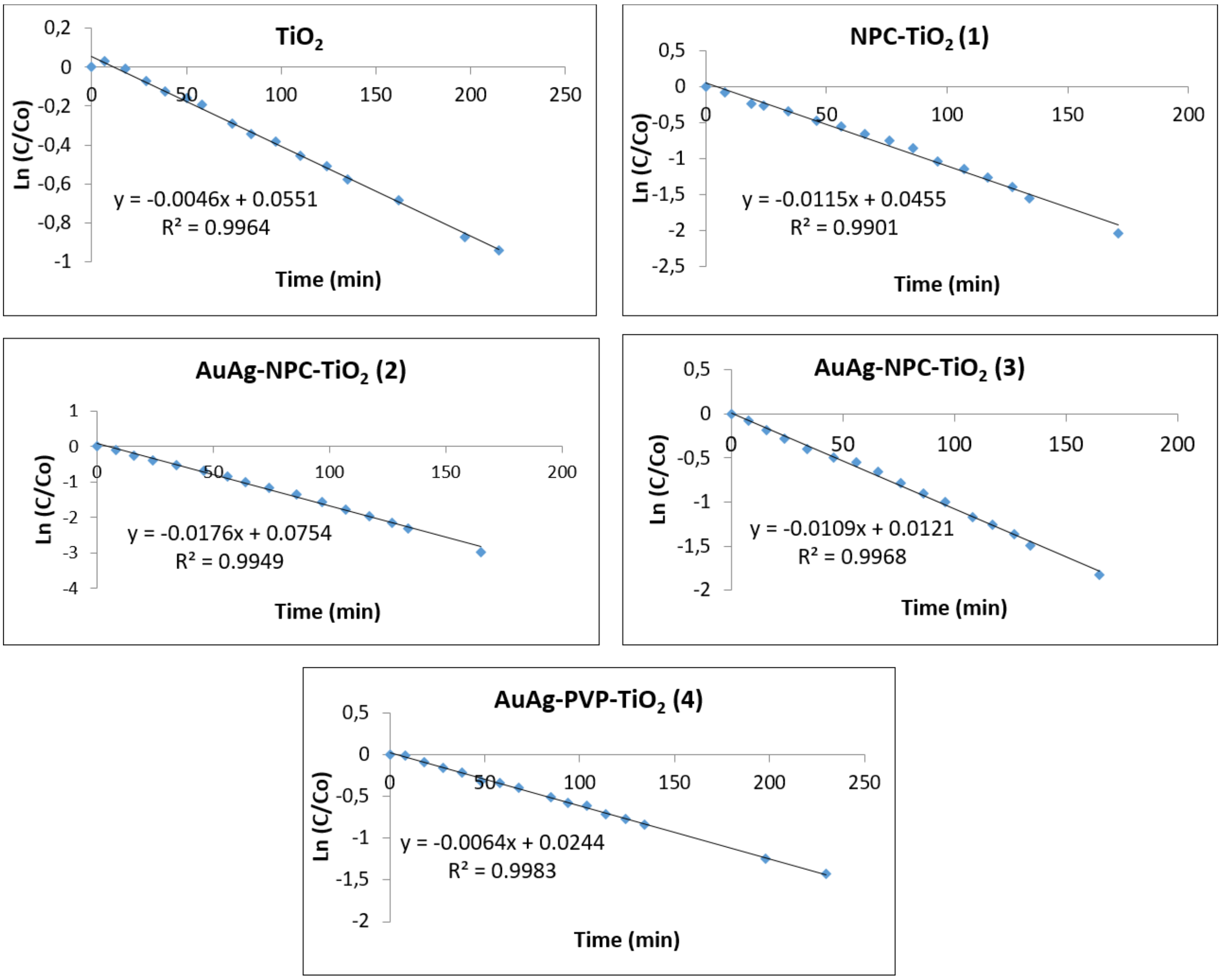

Figure S6. Kinetics of the photocatalysts **1**–**4** under visible light. The fitting results are represented assuming a pseudo-first order reaction.
